# Safety of lifitegrast: A real-world pharmacovigilance study based on FAERS

**DOI:** 10.1371/journal.pone.0321307

**Published:** 2025-04-24

**Authors:** Chu Zhang, Hui Wang, Xianglei Chen, Yong Liu, Puen Jiang

**Affiliations:** 1 Chizhou Aier Eye Hospital, Chizhou, Anhui, China; 2 Nanling County Hospital of Traditional Chinese Medicine, Wuhu, Anhui, China; 3 Anhui Aier Eye Hospital, Hefei, Anhui, China; 4 The First Affiliated Hospital of Chongqing Medical University, Chongqing, China; Auburn University, UNITED STATES OF AMERICA

## Abstract

To comprehensively assess the safety of Lifitegrast, a drug for dry eye disease, we conducted a comprehensive analysis of adverse event (AE) reports from the FDA Adverse Event Reporting System (FAERS). This retrospective study examined all AE reports submitted between Q3 2016 and Q2 2024, employing disproportionality analysis to identify safety signals associated with Lifitegrast. Out of a total of 13,326,934 AE reports indexed in the FAERS database, 12,393 listed Lifitegrast as the primary suspect drug. A total of 230 ocular AE signals were identified, with 104 of these AEs being detected by different analysis algorithms. Among these signals, 71 AEs were documented in the product insert of Lifitegrast, including but not limited to eye irritation, eye pain and eye swelling. AEs not mentioned in the drug labeling were identified, such as glaucoma and cataracts.AEs associated with Lifitegrast are more commonly observed in male patients and those aged over 65 years.The insights derived from this FAERS database analysis are significant for optimizing the use of Lifitegrast while minimizing potential side effects, thereby enhancing the safety of its clinical application.

## Introduction

Dry eye disease (DED) is a multifactorial chronic disorder of the ocular surface characterized by inflammation of the ocular surface and lacrimal glands, along with a reduction in tear quality or quantity [1–2]. It is estimated that approximately 6.8% of adults in the United States are affected by DED, and this prevalence significantly increases with age, as evidenced by a 2024 study that found a 40.0% prevalence rate among individuals aged 60–69 [3]. DED can cause symptoms such as eye irritation, stinging, dryness, and visual fatigue [4–5], significantly impacting patients’ quality of life [6] and imposing a substantial economic burden on individuals in Europe, North America, and Asia [7].

Given this situation, how to effectively treat and alleviate DED has become an important issue worthy of attention.Currently, the management of DED includes lifestyle modifications, supplementation with artificial tears, and pharmacological interventions, such as cyclosporine A ophthalmic emulsion, corticosteroids, and tetracyclines. In 2016, Lifitegrast entered the market as the first approved drug for both the signs and symptoms of DED. It is a novel small molecule integrin antagonist that blocks the interaction between ICAM-1 and LFA-1, thus interrupting the T-cell mediated inflammatory cycle [8]. Several clinical trials have demonstrated that Lifitegrast can significantly improve signs and symptoms of DED over a 12-week period and has shown favorable safety profiles during long-term use of up to one year, with the most common adverse reactions including decreased vision, pain at the site of administration, irritation at the site of administration, and reaction at the site of administration, mostly mild to moderate in nature [9]^.^Although there is a considerable amount of preclinical and clinical trial data supporting the efficacy and safety of Lifitegrast, information on its safety profile in real-world applications remains limited [10].

FAERS is a large database containing spontaneously reported AEs related to medical products, designed to support the FDA’s post-market surveillance of drugs and therapeutic biologics. It collects AE reports submitted voluntarily by healthcare professionals, patients, and pharmaceutical manufacturers [11]. As one of the largest global databases for monitoring drug-related AEs [12–14], it provides a rich resource for studying medication safety. We utilized the FAERS database to identify adverse reactions reported during the post-marketing use of Lifitegrast and conducted a disproportionality analysis of these reactions. The findings from this study provide a scientific basis for clinical medication use, thereby enhancing patient drug safety.

## Methods

### Data source and preprocessing

We conducted a retrospective disproportionality analysis of AE reports involving Lifitegrast from the FAERS database (Q3 2016–Q2 2024) to identify potential safety signals. The FAERS database is updated on a quarterly basis and encompasses seven distinct datasets, including demographic and administrative information (DEMO), drug-related data (DRUG), adverse drug reaction reports (REAC), patient outcomes (OUTC), sources of reports (RPSR), start and end dates of drug therapy (THER), and indications for drug administration (INDI). Initially, we accumulated 13,326,934 DEMO reports. Due to the ongoing updates required for the FAERS database, duplicate reports were inevitably present. Therefore, before conducting statistical analyses, we performed deduplication according to the guidelines provided by the FDA. The specific procedures were as follows: when the CASEID matched, we selected the record with the more recent FDA_DT; if both CASEID and FDA_DT were identical, we retained the record with the higher PRIMARYID value [15–16]. After the deduplication process, which eliminated incomplete, erroneous, and duplicate reports, we obtained a dataset comprising 11,380,534 unique DEMO reports, 43,872,480 DRUG records, and 33,164,606 REAC entries.

**Fig 1:**
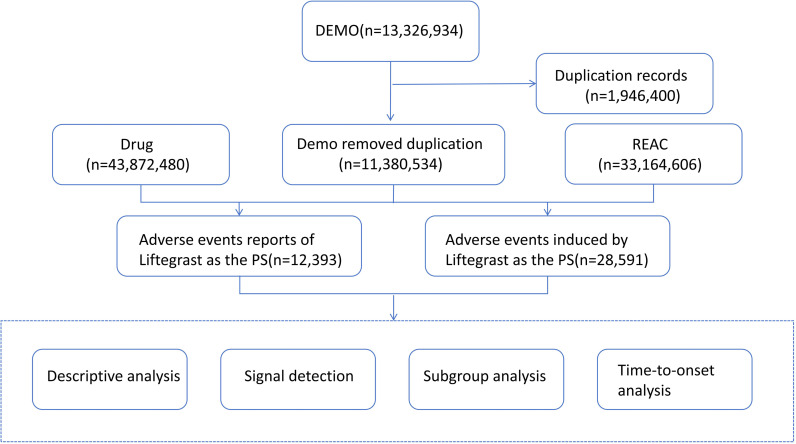
The process of selecting Liftegrast-associated AEs from FAERS database.

For this study, we selected AE reports where “Lifitegrast” or its brand name “XIIDRA” was designated as the primary suspect drug. To ensure the accuracy of the analysis, only AE reports that specified Lifitegrast as the primary suspect (PS) were included. Fig 1 presents the flow chart of the investigation process.This study is based on analysis of publicly available data from the FAERS database, which does not require individual patient consent as it consists of anonymized reports.Given that the data are already de-identified and publicly accessible, no further ethical approval or consent procedures were necessary.

### Data standardization and analysis

Each record in the FAERS database was coded according to the Medical Dictionary for Regulatory Activities (MedDRA) preferred terms (PT). We utilized these PTs to identify AE signals. During the data preprocessing phase, records containing inaccurate or missing dates, especially those with an event date (EVENT_DT) earlier than the start date (START_DT), were excluded to maintain data integrity. All data were classified and coded according to MedDRA PTs. To define specific health impacts, AEs were aggregated into standardized MedDRA queries (SMQs).

Disproportionality analysis was performed using reporting odds ratio (ROR) [17], proportional reporting ratio (PRR) [18], and Bayesian Confidence Propagation Neural Network (BCPNN) [19] to detect AE signals. These methods were applied to determine whether there was a significant association, i.e., whether there was an excess frequency of reports involving Lifitegrast and specific AEs. Detailed two-by-two contingency tables are provided in S1 Table. The formulas and thresholds for these disproportionality analyses are outlined in S2 Table.

Time-to-onset (TTO) was defined as the interval between the start date of treatment (START_DT) and the onset date of the AE (EVENT_DT). The distribution of TTO was described using medians and interquartile ranges, and comparisons were made regarding the distribution of AEs stratified by gender and age groups, as well as the cumulative incidence of AEs.

The Weibull shape parameter (WSP) test was employed to assess data regarding the TTO. The WSP test has been previously put forward as a technique for ascertaining the fluctuating incidence rate of AEs, which is contingent upon the drug’s mechanism of action and typically exhibits temporal variation [20–22]. Furthermore, the hazard functions associated with AEs can likewise be appraised through the application of a Weibull model to the time-to-event onset data [23]. Based on two observation metrics, namely the shape parameter β and the 95% confidence interval (CI), the WSP test categorizes the temporal variation in hazard occurrence into three distinct types. The initial category is the early failure-type profile (β < 1, 95% CI < 1), which signifies that the hazard escalates during the early phase of treatment and subsequently diminishes over time. The second category constitutes the random failure-type profile (β is equal to or approximately 1, with the 95% CI encompassing the value 1), which denotes a consistent hazard occurrence throughout the treatment period. The third category represents the wear-out failure-type profile (β > 1, 95% CI excludes the value 1), which indicates an accelerating hazard occurrence rate [20–22]. Subsequently, the Kaplan–Meier method was utilized to generate cumulative incidence curves of AEs associated with Lifitegrast, and the group differences were analyzed using a log-rank test, with a p-value threshold of less than 0.05 being considered indicative of statistical significance.

Subgroup analyses were conducted to examine the occurrence of AEs in different patient subgroups based on age (18–65 years, over 65 years) and gender (male and female).

This study utilized Microsoft Excel 2016 for table creation and R Studio (version 1.4.1717) for graph plotting and statistical analysis.

## Results

### Descriptive analysis

Table 1 summarizes the main clinical characteristics of Lifitegrast-related AE reports in the FAERS database from the third quarter of 2016 to the second quarter of 2024. A total of 12,393 reports were retrieved. In terms of gender distribution, male patients accounted for 9,128 reports (73.7%), female for 1,912 reports (15.4%), while the remaining 1,353 reports lacked gender information. In terms of patient age, the distribution was as follows: 19 cases (<18 years, 0.2%), 1,385 cases (18–64 years, 11.2%), and 1,585 cases (≥65 years, 12.8%). The remaining 9,404 cases (75.9%) did not provide age information. Regarding weight, 111 patients weighed less than 50 kg (0.9%), 85 patients weighed more than 100 kg (0.7%), and 1,231 patients weighed between 50 and 100 kg (9.9%), with the remainder (10,966 cases, 88.5%) having missing weight information.

**Table 1:** Clinical characteristics of Lifitegrast AE reports from the FAERS database (Q3 2016-Q2 2024).

Characteristics	Case number	Case proportion (%)
Number of reports	12393	
**Gender**		
Male	9128	73.7
Female	1912	15.4
Missing	1353	10.9
**Age, years**		
<18	19	0.2
18–64	1385	11.2
>=65	1585	12.8
Missing	9404	75.9
**Weight, kg**		
<50 kg	111	0.9
>100 kg	85	0.7
50~100 kg	1231	9.9
Missing	10966	88.5
**Reported Countries**		
United States	11203	90.4
Other countries	1187	9.5
**Reporter**		
Consumer	8844	71.4
Healthcare professional	426	3.4
Physician	2390	19.3
Pharmacist	206	1.7
Other Professional	433	3.5
Missing	94	0.8

Most reports originated from the United States (11,203 cases, 90.4%), with the rest coming from other countries (1,187 cases, 9.5%). Reports were mainly submitted by consumers (8,844 cases, 71.4%), followed by physicians (2,390 cases, 19.3%), with fewer reports from other healthcare professionals such as pharmacists.

**Fig 2:**
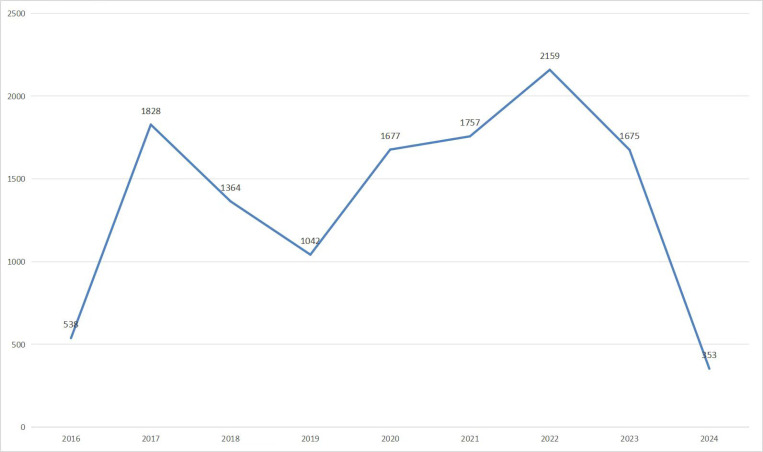
The annual distribution of drug-related AE reports associated with Lifitegrast.

Fig 2 illustrates the annual distribution of drug-related AE reports associated with Lifitegrast. The year with the lowest number of reports was 2024, with a total of 353 reports, whereas the highest number was seen in 2022, with 2,159 reports. Notably, from 2019 to 2022, there was a clear upward trend in the number of AE reports, which remained at a high level in 2023.

### Signal detection at the System Organ Class(SOC) level

AEs associated with Lifitegrast covered all 27 SOCs. Apart from eye disorders, the top five SOCs were general disorders and administration site conditions, injury, poisoning and procedural complications, nervous system disorders, product issues, and respiratory, thoracic, and mediastinal disorders. The summary of SOC signal strength associated with Lifitegrast is presented in Table 2.

**Table 2:** Signal strength of Lifitegrast AEs across SOCs from the FAERS database.

SOC	Case number	ROR (95%CI)	PRR (^2^)	IC(IC025)
Eye disorders^**^	10935	31.54 (30.79–32.3)	19.87 (196509.43)	4.29 (2.62)
General disorders and administration site conditions^*^	6649	1.41 (1.37–1.45)	1.31 (598.62)	0.39 (−1.27)
Injury, poisoning and procedural complications	2444	0.73 (0.7–0.76)	0.75 (228.03)	−0.41 (−2.08)
Nervous system disorders^*^	2361	1.07 (1.03–1.12)	1.07 (10.5)	0.09 (−1.57)
Product issues^*^	1055	2.09 (1.96–2.22)	2.05 (575.64)	1.03 (−0.63)
Respiratory, thoracic and mediastinal disorders	902	0.68 (0.63–0.72)	0.69 (135.72)	−0.54 (−2.21)
Gastrointestinal disorders	786	0.32 (0.29–0.34)	0.33 (1131.27)	−1.58 (−3.24)
Skin and subcutaneous tissue disorders	660	0.39 (0.36–0.42)	0.4 (633.09)	−1.32 (−2.99)
Infections and infestations	639	0.39 (0.36–0.43)	0.41 (580.75)	−1.29 (−2.96)
Immune system disorders^*^	496	1.42 (1.3–1.55)	1.41 (60.08)	0.5 (−1.17)
Psychiatric disorders	373	0.23 (0.21–0.26)	0.24 (937.21)	−2.05 (−3.71)
Musculoskeletal and connective tissue disorders	342	0.22 (0.2–0.25)	0.23 (907.85)	−2.1 (−3.76)
Ear and labyrinth disorders^*^	213	1.71 (1.5–1.96)	1.71 (62.7)	0.77 (−0.89)
Investigations	212	0.12 (0.11–0.14)	0.13 (1341.25)	−2.97 (−4.63)
Cardiac disorders	90	0.15 (0.12–0.18)	0.15 (431.55)	−2.71 (−4.38)
Social circumstances	87	0.67 (0.55–0.83)	0.67 (13.68)	−0.57 (−2.23)
Vascular disorders	84	0.15 (0.12–0.19)	0.15 (400.9)	−2.71 (−4.37)
Neoplasms benign, malignant and unspecified (incl cysts and polyps)	82	0.09 (0.07–0.11)	0.09 (780.52)	−3.47 (−5.14)
Metabolism and nutrition disorders	63	0.11 (0.08–0.14)	0.11 (468.34)	−3.2 (−4.86)
Surgical and medical procedures	35	0.08 (0.06–0.12)	0.09 (346.51)	−3.54 (−5.21)
Renal and urinary disorders	27	0.05 (0.03–0.07)	0.05 (530.55)	−4.41 (−6.07)
Congenital, familial and genetic disorders	16	0.21 (0.13–0.33)	0.21 (49.24)	−2.28 (−3.95)
Endocrine disorders	15	0.2 (0.12–0.33)	0.2 (48.75)	−2.33 (−4)
Reproductive system and breast disorders	10	0.05 (0.03–0.09)	0.05 (182.64)	−4.33 (−5.99)
Blood and lymphatic system disorders	10	0.02 (0.01–0.04)	0.02 (465.86)	−5.58 (−7.24)
Hepatobiliary disorders	4	0.02 (0.01–0.04)	0.02 (231.97)	−5.89 (−7.56)
Pregnancy, puerperium and perinatal conditions	1	0.01 (0–0.06)	0.01 (108.38)	−6.78 (−8.45)

Abbreviation: two asterisks (

**) indicate statistically significant signals in all algorithms; one asterisk (

*) indicates a statistically significant signal in at least one of the algorithms.ROR, reporting odds ratio; PRR, proportional reporting ratio; IC, information component; IC025, the lower limit of the 95% CI of the IC; CI, confidence interval; AEs, adverse events.

### Signal detection at the PT level

Table 3 lists the top 30 most frequent positive AEs for Lifitegrast at the PT level from the FAERS database. Among the AE signals associated with Lifitegrast, the most frequent positive AEs included eye irritation, vision blurred, eye pain, and ocular hyperaemia. AEs not mentioned in the product insert include hypersensitivity and hypoacusis.

**Table 3:** Top 30 most frequent positive AEs for Lifitegrast at the PT level from the FAERS database.

SOC	PT	Case number	ROR (95%CI)	PRR (2)	IC(IC025)
Eye disorders	Eye irritation	2245	104.46 (99.89–109.24)	96.35 (195730.42)	6.48 (4.81)
	Vision blurred	2237	43.79 (41.91–45.76)	40.45 (83326.12)	5.29 (3.62)
	Eye pain	923	39.57 (37.02–42.29)	38.32 (32504.83)	5.21 (3.55)
	Ocular hyperaemia	514	24.96 (22.86–27.26)	24.53 (11369.88)	4.59 (2.92)
	Lacrimation increased	475	35.14 (32.05–38.53)	34.57 (15044.69)	5.07 (3.4)
	Visual impairment	468	7.74 (7.06–8.48)	7.63 (2682.34)	2.92 (1.26)
	Eye pruritus	358	23.33 (21–25.92)	23.05 (7407.92)	4.5 (2.83)
	Eye discharge	308	68.45 (60.99–76.83)	67.73 (19132.87)	6 (4.33)
	Eye disorder	285	19.35 (17.2–21.76)	19.16 (4829.46)	4.24 (2.57)
	Eye swelling	190	11.89 (10.31–13.73)	11.82 (1864.41)	3.55 (1.88)
	Ocular discomfort	171	31.22 (26.81–36.36)	31.04 (4842.56)	4.92 (3.25)
	Foreign body sensation in eyes	110	39.13 (32.35–47.33)	38.98 (3938.59)	5.24 (3.57)
	Blindness	96	5.26 (4.3–6.43)	5.24 (328.38)	2.39 (0.72)
	Photophobia	95	11.64 (9.5–14.25)	11.6 (911.5)	3.52 (1.86)
General disorders and administration site conditions	Instillation site pain	1280	2862.13 (2586.8–3166.75)	2734.17 (1040381.86)	9.67 (8)
	Instillation site reaction	1019	33062.17 (23811.24–45907.19)	31885.08 (1138377.01)	10.13 (8.46)
	Instillation site irritation	240	787.1 (667.97–927.48)	780.51 (111605.49)	8.87 (7.2)
	Instillation site erythema	209	1050.64 (871.2–1267.04)	1042.97 (114456.81)	9.1 (7.43)
	Instillation site pruritus	187	1816.02 (1443.54–2284.61)	1804.16 (131729.3)	9.46 (7.79)
	Instillation site lacrimation	179	3475.69 (2593.94–4657.17)	3453.96 (155122.52)	9.76 (8.08)
	Instillation site discharge	118	4286.88 (2900.08–6336.83)	4269.21 (107420.29)	9.83 (8.15)
Nervous system disorders	Dysgeusia	1002	34.49 (32.35–36.77)	33.32 (30564.16)	5.02 (3.35)
	Taste disorder	173	14.05 (12.09–16.33)	13.97 (2059.62)	3.79 (2.12)
	Burning sensation	161	5.45 (4.66–6.36)	5.42 (578.24)	2.43 (0.77)
Immune system disorders	Hypersensitivity	325	3.65 (3.27–4.08)	3.62 (617.42)	1.85 (0.19)
Product issues	Product quality issue	320	6.44 (5.77–7.2)	6.38 (1447.37)	2.67 (1)
	Product container issue	199	44.91 (38.96–51.77)	44.61 (8169.75)	5.43 (3.76)
	Product packaging quantity issue	122	21.73 (18.16–26)	21.64 (2357.76)	4.41 (2.74)
Injury, poisoning and procedural complications	Product use complaint	243	26.3 (23.14–29.88)	26.08 (5733.73)	4.67 (3.01)
Ear and labyrinth disorders	Hypoacusis	94	3.71 (3.03–4.55)	3.71 (185.23)	1.89 (0.22)

Abbreviation: ROR, reporting odds ratio; PRR, proportional reporting ratio; IC, information component; IC025, the lower limit of the 95% CI of the IC; CI, confidence interval; PT, preferred term.

### Ocular AE signals

A total of 104 positive ocular AE signals associated with Lifitegrast were identified. Among these, 71 were known AEs that had been previously recorded in the product insert, while the remaining 33 were newly discovered AEs that had not been previously documented. Table 4 lists the top 30 unexpected ocular AE signals, including cataract and glaucoma. These newly identified AEs provide important clues for subsequent drug safety assessments.

**Table 4:** The top 30 unexpected ocular AEs at the PT level for Lifitegrast.

SOC	PT	Case number	ROR (95%CI)	PRR (^2^)	IC(IC025)
Eye disorders	Cataract	88	3.21 (2.6–3.95)	3.2 (132.82)	1.68 (0.01)
	Glaucoma	48	5.52 (4.16–7.34)	5.52 (176.67)	2.46 (0.79)
	Asthenopia	47	18.31 (13.72–24.43)	18.28 (755.79)	4.17 (2.5)
	Madarosis	43	5.48 (4.06–7.39)	5.47 (156.48)	2.45 (0.78)
	Periorbital swelling	39	12.34 (9–16.91)	12.32 (401.41)	3.61 (1.94)
	Macular degeneration	33	6.31 (4.48–8.89)	6.31 (146.61)	2.65 (0.98)
	Corneal abrasion	27	48.66 (33.1–71.53)	48.61 (1208.44)	5.55 (3.88)
	Dacryostenosis acquired	25	37.82 (25.39–56.33)	37.79 (867.03)	5.19 (3.53)
	Vitreous floaters	25	5.48 (3.7–8.12)	5.48 (91.06)	2.45 (0.78)
	Lacrimation decreased	13	68.76 (39.29–120.32)	68.72 (819)	6.02 (4.34)
	Eye movement disorder	11	4.07 (2.25–7.36)	4.07 (25.36)	2.02 (0.35)
	Photopsia	11	4.52 (2.5–8.17)	4.52 (30.03)	2.17 (0.5)
	Corneal scar	10	48.66 (25.84–91.62)	48.64 (447.84)	5.55 (3.87)
	Metamorphopsia	10	13.16 (7.06–24.55)	13.16 (111.06)	3.7 (2.03)
	Halo vision	8	17.58 (8.74–35.34)	17.57 (123.19)	4.11 (2.44)
	Myopia	7	7.04 (3.35–14.81)	7.04 (36.06)	2.81 (1.14)
	Meibomian gland dysfunction	7	29.8 (14.07–63.11)	29.8 (189.91)	4.86 (3.19)
	Glare	7	34.2 (16.13–72.54)	34.2 (219.1)	5.05 (3.38)
	Eyelash changes	5	45.95 (18.8–112.33)	45.94 (211.43)	5.47 (3.78)
	Strabismus	5	4.34 (1.8–10.45)	4.34 (12.82)	2.11 (0.45)
	Scleral discolouration	4	14.99 (5.59–40.19)	14.99 (51.55)	3.89 (2.21)
	Corneal opacity	4	6.96 (2.61–18.61)	6.96 (20.31)	2.79 (1.12)
	Eyelid function disorder	4	14.8 (5.52–39.68)	14.8 (50.8)	3.87 (2.2)
	Swollen tear duct	4	85.77 (31.06–236.84)	85.76 (311.98)	6.32 (4.61)
	Astigmatism	4	5.85 (2.19–15.62)	5.85 (15.99)	2.54 (0.87)
	Corneal exfoliation	3	69.47 (21.67–222.75)	69.47 (190.98)	6.04 (4.32)
	Corneal thinning	3	28.01 (8.91–88.05)	28.01 (76.29)	4.77 (3.09)
	Corneal defect	3	48.24 (15.2–153.13)	48.24 (133.23)	5.53 (3.84)
	Presbyopia	3	15.04 (4.81–46.97)	15.04 (38.8)	3.89 (2.22)
	Dermatochalasis	3	33.4 (10.6–105.26)	33.4 (91.64)	5.02 (3.33)

Abbreviation: ROR, reporting odds ratio; PRR, proportional reporting ratio; IC, information component; IC025, the lower limit of the 95% CI of the IC; CI, confidence interval; PT, preferred term.

### Subgroup analysis

From a subgroup perspective, the results indicated that the number of AEs was higher in male patients than in females. Among the top ten ROR signal strength-ranked related AEs, male patients using Lifitegrast experienced AEs such as vision blurred, eye irritation, eye pain, and increased lacrimation. Female patients using Lifitegrast experienced major AEs including eye irritation, vision blurred, eye pain, and ocular hyperaemia. Age-stratified analysis found that elderly patients (≥65 years) had more Lifitegrast-related AEs. The main AEs in patients aged 18–65 years included dysgeusia, headache, taste disorder, instillation site pain, and instillation site reaction. For elderly patients (>65 years), the major side effects of Lifitegrast included vision blurred, eye irritation, eye pain, and increased lacrimation. Specific details can be seen in S3-S6 Tables.

### TTO analysis

**Fig 3:**
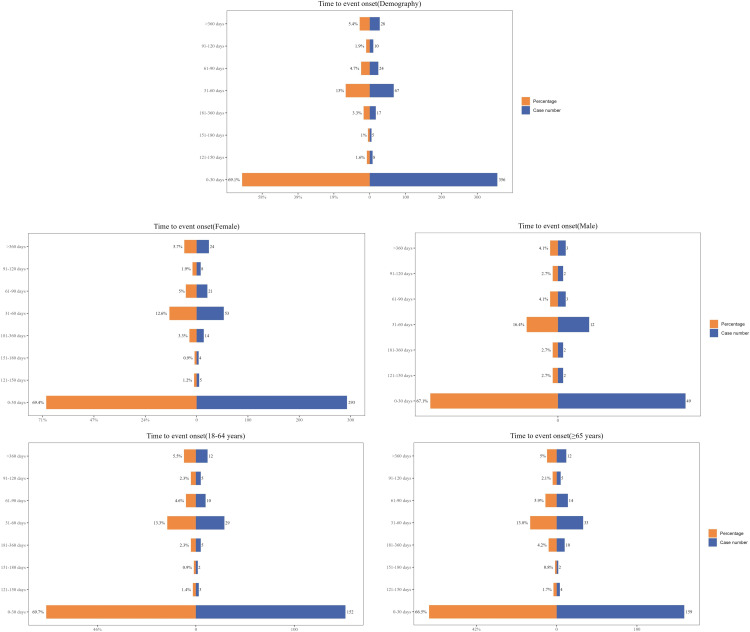
Time to onset of Lifitegrast-related AEs.

**Fig 4:**
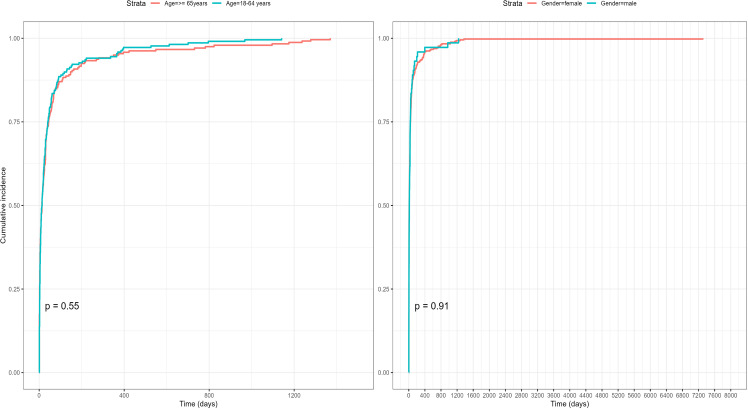
The distribution of Lifitegrast-related AE onset times across gender and age groups.

The onset time of AEs was calculated by subtracting the initial Lifitegrast use date from the AE occurrence date in the reports. The study results indicated that the median onset time of AEs was 12 days after starting Lifitegrast, with 69.1% (356 cases) of AEs occurring within 30 days of initiation. Weibull distribution analysis suggested an early failure pattern.Furthermore, we examined the distribution of the onset time of AEs following Lifitegrast use across different gender and age groups.However, analysis using the Kaplan-Meier method revealed no statistically significant differences. In all subgroups, Weibull distribution similarly indicated an early failure pattern, suggesting that AEs predominantly occurred in the initial stages of drug use. More detailed information can be found in Figs 3–4 and Table 5.

**Table 5:** Time to onset of Lifitegrast -associated AEs and Weibull distribution analysis.

Group		Case number	TTO (days)	Weibull distribution		
			Median (IQR)	Scale parameter: α(95%CI)	Shape parameter: β(95%CI)	Type
Demography		515	12 (2–40)	31.3(25.7–37.0)	0.51(0.48–0.54)	Early failure
Gender	Female	422	11 (8–15)	31.0(24.8–37.3)	0.50(0.47–0.53)	Early failure
	Male	73	13 (8–30)	32.3(18.40–46.2)	0.57 (0.48–0.66)	Early failure
Age, years	18–64	218	13.5 (9–19)	30.9(23.16–38.71)	0.56 (0.51–0.61)	Early failure
	>=65	239	13 (9–20)	32.7(24.2–41.3)	0.52(0.47–0.57)	Early failure

Abbreviation: TTO, time to onset; CI, confidence interval; IQR, interquartile range.

## Discussion

Previous studies on Lifitegrast have lacked substantial real-world evidence. In contrast, the FAERS database, with its 12,393 AE reports, offers a more comprehensive assessment of safety and risk profiles compared to the 1,401 participants in clinical trials. It is also crucial to highlight that information derived from clinical trials may not accurately reflect real-world conditions.Therefore, this study addresses this gap by conducting an in-depth analysis of the FAERS database, providing clinicians with a basis for the rational use of Lifitegrast and aiding them in identifying and managing potential risks promptly.

Our analysis indicates that male patients reported a greater number of AEs, potentially due to factors such as their higher tendency towards unhealthy lifestyle choices like smoking and exposure to secondhand smoke. Smoking is a known risk factor for DED [24], which can manifest as ocular discomfort, including symptoms like ocular hyperemia and irritation. Similarly, secondhand smoke exposure may induce eye irritation [25]—symptoms that patients might report as Lifitegrast-related AEs. Additionally, Lifitegrast-related AEs were more common in patients over 65 years old, aligning with prior research that indicates aging is associated with meibomian gland atrophy, leading to increased tear evaporation and a higher risk of DED [26–28].This suggests that as the clinical application of Lifitegrast increases, it is crucial for clinicians to remain vigilant about the potential adverse reactions associated with Lifitegrast, particularly in male and elderly patients. Approximately 95.4% of AE reports were submitted by consumers, highlighting their role in pharmacovigilance and drug utilization. Reports were predominantly from the United States, which may be indicative of the higher level of awareness and easier access to the FAERS reporting system available to patients and healthcare providers in this country. According to the annual report distribution, Lifitegrast AE reports peaked notably in 2017 and again in 2022, with the latter marking the highest point. From 2019 through 2022, there was a clear upward trend in the number of reports, which remained at a high level into 2023.This trend can be attributed to several factors. The increase in 2017 primarily reflected the initial phase after Lifitegrast’s market launch, during which heightened market promotion and growing patient awareness led to increased usage. Additionally, doctors, inexperienced with the drug’s side effects, were more proactive in reporting to gather data. Between 2019 and 2022, as the number of long-term users grew and both healthcare providers and patients gained a deeper understanding of the medication, the efficiency of identifying and reporting adverse reactions improved, driving a sustained rise in reported cases. The peak in 2022 was further bolstered by enhanced market education, expanded insurance coverage, and relatively limited market competition, allowing more patients access to the drug. By 2023, despite new competing drugs entering the market, Lifitegrast maintained a significant user base and efficacy advantage, preserving its market share and thus sustaining a high volume of adverse reaction reports.It should be noted that due to missing data, our results can only be considered indicative, yet they underscore the importance of monitoring AEs for Lifitegrast.

The study identified several common ocular AEs following the use of Lifitegrast, including eye irritation, vision blurred, eye pain, and ocular hyperaemia. Previous clinical studies corroborate these findings. For instance, Donnenfeld et al.‘s [29] one-year safety study (SONATA) showed that 15.0% of patients experienced instillation site irritation (such as burning), 13.2% had instillation site reactions (such as redness and swelling), and 11.4% reported decreased vision. Similarly, Tauber et al.’s [30] study (OPUS-2) reported comparable AEs. The mechanisms of these AEs may be linked to the transient physical irritation caused by lifitegrast as an ocular solution upon instillation, such as the cooling effect or reflex responses due to contact with the cornea or conjunctiva. Vision blurred might be attributed to temporary changes in tear film stability induced by the eye drops, affecting visual clarity. However, it is worth noting that these AEs often resolve within minutes after instillation [29].

Our study also identified significant AEs, including newly discovered ocular AEs such as cataracts and glaucoma, as well as systemic AEs like hypersensitivity and hypoacusis. While Lifitegrast is used for the treatment of DED, its association with cataracts and glaucoma remains unclear. Given that DED [31–32], cataracts [33], and glaucoma [34–35]are all age-related conditions, it is likely that older adults may represent a larger proportion of patients receiving Lifitegrast for dry eye.The study found that individuals aged 65 years and older reported more AEs compared to younger patients. This population is more susceptible to age-related diseases, and we speculate that the occurrence of cataracts and glaucoma following Lifitegrast treatment in these patients could be due to age-induced lens opacity and shallowing of the anterior chamber. However, our findings suggest that close monitoring of ocular health is necessary for dry eye patients undergoing Lifitegrast therapy. If symptoms such as vision loss or ocular discomfort arise, cataracts and glaucoma should be considered. Regular slit-lamp examinations, intraocular pressure measurements, assessments of anterior chamber depth, and gonioscopy are recommended to promptly identify risks of cataracts and glaucoma.Hypersensitivity could be related to abnormal immune responses triggered by drug components, causing an excessive immune response. hypoacusis has not been previously reported, so its mechanism warrants further investigation.

Table 1 shows varying rates of AEs between genders. When evaluating drug safety, performing gender-specific analyses is essential, considering observed gender disparities [36]. From a subgroup analysis perspective, our study reveals the impact of gender and age on the occurrence of AEs following Lifitegrast use. Notably, the proportion of AEs differed by gender, with males reporting more AEs than females. In the top five AEs ranked by relative risk signal strength, males treated with Lifitegrast primarily reported visual impairment, uveitis, vision blurred, vitritis, and eye inflammation, similar to females. Compared to women, men tend to wait for symptoms to resolve without intervention, engaging less in health-promoting behaviors and interacting less with healthcare professionals, which might contribute to occur more frequently AEs [37]. Further age-stratified analysis revealed Lifitegrast-related AEs occur more frequently in patients over 65 years old. Specifically, in younger patients aged 18–64 years, the most common AEs after Lifitegrast use were visual impairment, vision blurred, blindness, cataracts, and uveitis. For elderly patients above 65 years, common AEs included pruritus, rash, psoriasis, acne, and erythema. This phenomenon can be attributed to several factors. Firstly, the elderly often have a higher prevalence of comorbidities, such as cardiovascular diseases and diabetes, which can interact with Lifitegrast and increase the risk of AEs. For instance, a systematic review and meta-analysis found that elderly patients with multiple comorbidities were more susceptible to drug-drug interactions [38]. Secondly, age-related physiological changes, such as decreased renal function and altered drug metabolism, can affect the pharmacokinetics and pharmacodynamics of Lifitegrast, making elderly patients more susceptible to AEs. For example, reduced renal clearance in older adults may lead to increased drug accumulation, potentially exacerbating skin conditions like psoriasis and acne [39]. Additionally, cognitive decline in the elderly may impact their medication adherence, as they might forget to take their medication or take it incorrectly, further contributing to more AEs [40].

The study also analyzed the temporal trends of AEs associated with Lifitegrast using Weibull distribution models to predict the timing of these events, aiding in establishing effective monitoring timelines for drug-related AEs. According to the Weibull distribution analysis, the median onset time of AEs was 12 days (IQR 2–40 days) among all 515 cases, indicating an early failure type pattern. Gender-wise, the median onset time for females was 11 days (IQR 8–15 days), and for males, it was 13 days (IQR 8–30 days). Age-stratified analysis showed that the median onset time for patients aged 18–64 years was 13.5 days (IQR 9–19 days), and for those 65 years and older, it was 13 days (IQR 9–20 days). These findings emphasize the importance of close monitoring of AEs early in treatment, particularly within the first month, aiding in improving patient safety and treatment outcomes. It is noteworthy that, due to symptom improvement, some patients may discontinue the drug prematurely, resulting in a concentration of AEs within the first month. Understanding the relationship between medication duration and onset timing is crucial for assessing drug safety, identifying specific risk windows, and helping prevent or diagnose AEs early [41].

This study contributes valuable insights by highlighting the need for timely alleviation of discomfort and improving treatment effectiveness through better understanding of the temporal distribution of AEs associated with Lifitegrast use.

Limitations of our study include the inherent limitations of using the FAERS database for analysis. While we can identify signals of correlation between drugs and AEs, the limited information in the FAERS database makes it challenging to rule out the influence of other potential variables such as concomitant medications or the natural course of diseases, complicating causal inference from these data. Furthermore, as FAERS data primarily originate from Western countries (e.g., U.S., Canada) with limited representation of Asian populations, our findings may not fully capture regional pharmacovigilance patterns in Asia. This underrepresentation underscores the need for continued monitoring of adverse events in FAERS, particularly from underserved demographics. Future research should leverage both FAERS and national pharmacovigilance systems like Japanese Adverse Event Reporting System to generate region-specific real-world evidence addressing this geographic disparity.Additionally, the reliance on voluntary reporting in the FAERS database introduces issues of incomplete or inaccurate reporting and possible delays or omissions, which may introduce biases affecting the accuracy of AE measurements. A significant limitation is also that the FAERS does not provide background information on the number of patients taking the medication. Without a medical background, individuals may find it challenging to understand the medical terminology involved in FAERS, potentially leading to difficulties in accurately describing their symptoms. This can adversely affect the quality of reports and our analysis. Caution is needed when interpreting the results, and more clinical evidence is required to validate the AEs signals captured in this study.

## Summary

Through pharmacoepidemiological analysis utilizing the FAERS database, we identified ocular and systemic AE signals associated with Lifitegrast use in DED treatment, along with the temporal distribution characteristics of these AEs. Our study corroborated previously documented AEs such as eye irritation, eye pain, and eye swelling, while also uncovering novel ocular AEs including cataracts and glaucoma. Additionally, more AEs were reported among male patients and individuals aged 65 years or older.These findings underscore the importance of personalized monitoring for different genders and age groups in clinical practice and suggest that gender and age should be taken into account when formulating treatment plans. Although our research provides valuable evidence on the safety profile of Lifitegrast, it is important to acknowledge the inherent limitations of the FAERS database, particularly the potential impact on report quality and our analysis due to the majority of AE reports being submitted by patients. Future research should incorporate longer follow-up periods for monitoring AEs related to Lifitegrast. Prospective cohort studies and long-term clinical observations are warranted to confirm the phenomena and conclusions derived from this study.

## Supporting information

S1 Table Two-by-two contingency table for disproportionality analyses. (DOCX)

S2 Table Major algorithms used for signal detection. (DOCX)

S3 Table Top 30 most frequent AEs for Lifitegrast at the PT level in males from the FAERS database. (DOCX)

S4 Table Top 30 most frequent AEs for Lifitegrast at the PT level in female from the FAERS database. (DOCX)

S5 Table Top 30 most frequent AEs for Lifitegrast at the PT level in patients aged 18–65 from the FAERS database. (DOCX)

S6 Table Top 30 most frequent AEs for Lifitegrast at the PT level in patients aged over 65 from FAERS the database. (DOCX)
