# Supplementary material for: Safety of lifitegrast: A real-world pharmacovigilance study based on FAERS
Source: PLoS One. 2025 Apr 24;20(4):e0321307. doi: 10.1371/journal.pone.0321307 (PMC12021224; doi:10.1371/journal.pone.0321307)
Supplement: S1 Table — (DOCX) [file pone.0321307.s001.docx]

**S1 Table. Two-by-two contingency table for disproportionality analyses.**

|  | **Target AEs** | **Other AEs** | **Total** |
| --- | --- | --- | --- |
| Lifitegrast | a | b | a+b |
| Other drugs | c | d | c+d |
| Total | a+c | b+d | a+b+c+d |

Abbreviation: AEs, adverse events; a, number of reports containing both the Lifitegrast and target adverse drug reaction; b, number of reports containing other adverse drug reaction of the Lifitegrast; c, number of reports containing the target adverse drug reaction of other drugs; d, number of reports containing other drugs and other adverse drug reactions.
