# Supplementary material for: Safety of lifitegrast: A real-world pharmacovigilance study based on FAERS
Source: PLoS One. 2025 Apr 24;20(4):e0321307. doi: 10.1371/journal.pone.0321307 (PMC12021224; doi:10.1371/journal.pone.0321307)
Supplement: S2 Table — (DOCX) [file pone.0321307.s002.docx]

**S2 Table. Major algorithms used for signal detection.**

| **Algorithms** | **Equation** | **Criteria** |
| --- | --- | --- |
| ROR | ROR=ad/b/c | lower limit of 95% CI>1, N≥3 |
|  | 95%CI=e^ln(ROR)±1.96(1/a+1/b+1/c+1/d)^0.5^ |  |
| PRR | PRR=a(c+d)/c/(a+b) | PRR≥2, χ^2^≥4, N≥3 |
|  | χ^2^=[(ad-bc)^2](a+b+c+d)/[(a+b)(c+d)(a+c)(b+d)] |  |
| BCPNN | IC=log_2_a(a+b+c+d)(a+c)(a+b) | IC025>0 |
|  | 95%CI= E(IC) ± 2V(IC)^0.5 |  |

Abbreviation: a, number of reports containing both the Lifitegrast and target adverse drug reaction; b, number of reports containing other adverse drug reaction of the Lifitegrast; c, number of reports containing the target adverse drug reaction of other drugs; d, number of reports containing other drugs and other adverse drug reactions. 95%CI, 95% confidence interval; N, the number of reports; χ2, chi-squared; IC, information component; IC025, the lower limit of 95% CI of the IC; E(IC), the IC expectations; V(IC), the variance of IC.
