# Supplementary material for: Safety of lifitegrast: A real-world pharmacovigilance study based on FAERS
Source: PLoS One. 2025 Apr 24;20(4):e0321307. doi: 10.1371/journal.pone.0321307 (PMC12021224; doi:10.1371/journal.pone.0321307)
Supplement: S3 Table — (DOCX) [file pone.0321307.s003.docx]

**S3 Table. Top 30 most frequent AEs for Lifitegrast at the PT level in males from the FAERS database.**

| **SOC** | **PT** | **Case number** | **ROR (95%CI)** | **PRR (χ^2^)** | **IC(IC025)** |
| --- | --- | --- | --- | --- | --- |
| Eye disorders | Vision blurred | 442 | 72.36 ( 65.49 - 79.95 ) | 64.61 ( 27082.86 ) | 5.98 ( 4.31 ) |
|  | Eye irritation | 340 | 149.38 ( 133.34 - 167.35 ) | 136.98 ( 43721.65 ) | 7.03 ( 5.36 ) |
|  | Eye pain | 120 | 50.73 ( 42.24 - 60.93 ) | 49.26 ( 5576.62 ) | 5.6 ( 3.93 ) |
|  | Lacrimation increased | 74 | 44.74 ( 35.48 - 56.41 ) | 43.94 ( 3057.22 ) | 5.43 ( 3.77 ) |
|  | Ocular hyperaemia | 67 | 25.78 ( 20.23 - 32.86 ) | 25.37 ( 1555.1 ) | 4.65 ( 2.99 ) |
|  | Visual impairment | 66 | 8.66 ( 6.78 - 11.04 ) | 8.53 ( 438.26 ) | 3.09 ( 1.42 ) |
|  | Eye disorder | 42 | 24.17 ( 17.81 - 32.8 ) | 23.93 ( 915.2 ) | 4.57 ( 2.9 ) |
|  | Eye discharge | 33 | 62.94 ( 44.51 - 89.01 ) | 62.44 ( 1950.54 ) | 5.93 ( 4.26 ) |
|  | Eye pruritus | 31 | 18.42 ( 12.92 - 26.27 ) | 18.29 ( 503.57 ) | 4.18 ( 2.52 ) |
| General disorders and administration site conditions | Drug ineffective | 195 | 2.32 ( 2.01 - 2.68 ) | 2.26 ( 139.5 ) | 1.17 ( -0.49 ) |
|  | Instillation site reaction | 160 | 30190.08 ( 17766.68 - 51300.57 ) | 29002.7 ( 397730.15 ) | 11.28 ( 9.6 ) |
|  | Instillation site pain | 136 | 3135.82 ( 2450.99 - 4012.01 ) | 3031.02 ( 194801.05 ) | 10.49 ( 8.81 ) |
|  | Instillation site lacrimation | 29 | 7219.74 ( 3604.14 - 14462.43 ) | 7168.28 ( 57151.29 ) | 10.95 ( 9.22 ) |
|  | Instillation site irritation | 26 | 837.04 ( 538.9 - 1300.12 ) | 831.7 ( 16519.34 ) | 9.32 ( 7.63 ) |
| Injury, poisoning and procedural complications | Overdose | 113 | 5.13 ( 4.25 - 6.18 ) | 5.01 ( 364.28 ) | 2.32 ( 0.66 ) |
|  | Product dose omission issue | 64 | 2.9 ( 2.27 - 3.72 ) | 2.87 ( 78.48 ) | 1.52 ( -0.15 ) |
|  | Product use complaint | 41 | 37.3 ( 27.37 - 50.85 ) | 36.94 ( 1414.75 ) | 5.19 ( 3.52 ) |
|  | Incorrect dose administered | 36 | 2.27 ( 1.64 - 3.16 ) | 2.26 ( 25.44 ) | 1.18 ( -0.49 ) |
|  | Product use issue | 29 | 2.02 ( 1.4 - 2.92 ) | 2.02 ( 14.92 ) | 1.01 ( -0.65 ) |
|  | Accidental exposure to product | 25 | 3.5 ( 2.36 - 5.19 ) | 3.48 ( 44.3 ) | 1.8 ( 0.13 ) |
|  | Dysgeusia | 100 | 27.2 ( 22.29 - 33.21 ) | 26.56 ( 2438.3 ) | 4.72 ( 3.05 ) |
|  | Burning sensation | 28 | 9.11 ( 6.28 - 13.22 ) | 9.06 ( 200.2 ) | 3.17 ( 1.51 ) |
|  | Taste disorder | 25 | 13.38 ( 9.02 - 19.85 ) | 13.3 ( 283.25 ) | 3.73 ( 2.06 ) |
| Product issues | Product quality issue | 60 | 10.72 ( 8.3 - 13.84 ) | 10.57 ( 518.84 ) | 3.4 ( 1.73 ) |
|  | Product packaging quantity issue | 33 | 46.05 ( 32.6 - 65.05 ) | 45.69 ( 1418.78 ) | 5.49 ( 3.82 ) |
|  | Product container issue | 26 | 58.12 ( 39.36 - 85.82 ) | 57.76 ( 1420.12 ) | 5.82 ( 4.15 ) |
| Immune system disorders | Hypersensitivity | 37 | 4.42 ( 3.2 - 6.11 ) | 4.39 ( 96.92 ) | 2.13 ( 0.47 ) |

Abbreviation: ROR, reporting odds ratio; PRR, proportional reporting ratio; IC, information component; IC025, the lower limit of the 95% CI of the IC; CI, confidence interval; PT, preferred term.
