# Supplementary material for: Safety of lifitegrast: A real-world pharmacovigilance study based on FAERS
Source: PLoS One. 2025 Apr 24;20(4):e0321307. doi: 10.1371/journal.pone.0321307 (PMC12021224; doi:10.1371/journal.pone.0321307)
Supplement: S4 Table — (DOCX) [file pone.0321307.s004.docx]

**S4 Table. Top 30 most frequent AEs for Lifitegrast at the PT level in female from the FAERS database.**

| **SOC** | **PT** | **Case number** | **ROR (95%CI)** | **PRR (χ^2^)** | **IC(IC025)** |
| --- | --- | --- | --- | --- | --- |
| Eye disorders | Eye irritation | 1793 | 92.26 ( 87.71 - 97.04 ) | 84.76 ( 134868.13 ) | 6.27 ( 4.6 ) |
|  | Vision blurred | 1549 | 35.06 ( 33.26 - 36.95 ) | 32.64 ( 45824.73 ) | 4.97 ( 3.31 ) |
|  | Eye pain | 745 | 35.84 ( 33.26 - 38.61 ) | 34.65 ( 23396.9 ) | 5.06 ( 3.39 ) |
|  | Ocular hyperaemia | 401 | 23.37 ( 21.14 - 25.83 ) | 22.96 ( 8203.54 ) | 4.48 ( 2.82 ) |
|  | Visual impairment | 375 | 7.18 ( 6.48 - 7.96 ) | 7.07 ( 1944.32 ) | 2.81 ( 1.15 ) |
|  | Lacrimation increased | 364 | 31.98 ( 28.78 - 35.54 ) | 31.47 ( 10352.69 ) | 4.92 ( 3.26 ) |
|  | Eye pruritus | 304 | 22.13 ( 19.73 - 24.82 ) | 21.83 ( 5893.37 ) | 4.41 ( 2.75 ) |
|  | Eye discharge | 256 | 64.32 ( 56.6 - 73.09 ) | 63.58 ( 14654.43 ) | 5.89 ( 4.22 ) |
|  | Eye disorder | 225 | 17.72 ( 15.52 - 20.24 ) | 17.55 ( 3441.69 ) | 4.11 ( 2.44 ) |
|  | Eye swelling | 157 | 10.45 ( 8.93 - 12.24 ) | 10.39 ( 1316.4 ) | 3.36 ( 1.69 ) |
|  | Ocular discomfort | 145 | 31.57 ( 26.73 - 37.28 ) | 31.36 ( 4108.67 ) | 4.92 ( 3.25 ) |
| General disorders and administration site conditions | Instillation site pain | 933 | 2134.64 ( 1893.38 - 2406.65 ) | 2043.42 ( 552301.14 ) | 9.21 ( 7.54 ) |
|  | Drug ineffective | 838 | 1.68 ( 1.57 - 1.8 ) | 1.66 ( 223.15 ) | 0.73 ( -0.94 ) |
|  | Instillation site reaction | 662 | 27128.04 ( 17565.22 - 41897.02 ) | 26305.14 ( 535402.52 ) | 9.66 ( 7.99 ) |
|  | Instillation site irritation | 177 | 620.43 ( 510.69 - 753.75 ) | 615.41 ( 62489.89 ) | 8.47 ( 6.8 ) |
|  | Instillation site erythema | 140 | 768.48 ( 610.81 - 966.84 ) | 763.55 ( 55674.84 ) | 8.64 ( 6.97 ) |
|  | Condition aggravated | 135 | 1.15 ( 0.97 - 1.36 ) | 1.14 ( 2.47 ) | 0.19 ( -1.47 ) |
|  | Instillation site pruritus | 134 | 1372.04 ( 1042.03 - 1806.56 ) | 1363.62 ( 69266.89 ) | 9.02 ( 7.34 ) |
|  | Instillation site lacrimation | 123 | 2293.76 ( 1629.81 - 3228.19 ) | 2280.84 ( 75080.29 ) | 9.26 ( 7.58 ) |
| Nervous system disorders | Dysgeusia | 776 | 31.69 ( 29.46 - 34.09 ) | 30.6 ( 21460.17 ) | 4.89 ( 3.22 ) |
|  | Headache | 309 | 1.17 ( 1.05 - 1.31 ) | 1.17 ( 7.51 ) | 0.22 ( -1.44 ) |
|  | Taste disorder | 120 | 12.67 ( 10.57 - 15.18 ) | 12.6 ( 1263.42 ) | 3.64 ( 1.97 ) |
|  | Burning sensation | 117 | 4.34 ( 3.62 - 5.21 ) | 4.32 ( 297.56 ) | 2.11 ( 0.44 ) |
| Immune system disorders | Hypersensitivity | 252 | 3.15 ( 2.78 - 3.56 ) | 3.12 ( 363.28 ) | 1.64 ( -0.03 ) |
| Product issues | Product quality issue | 226 | 5.55 ( 4.87 - 6.33 ) | 5.5 ( 828.71 ) | 2.45 ( 0.79 ) |
|  | Product container issue | 138 | 38.1 ( 32.11 - 45.21 ) | 37.87 ( 4739.14 ) | 5.18 ( 3.51 ) |
| Injury, poisoning and procedural complications | Product dose omission issue | 193 | 1.54 ( 1.34 - 1.78 ) | 1.54 ( 36.27 ) | 0.62 ( -1.05 ) |
|  | Product use complaint | 184 | 25.65 ( 22.14 - 29.72 ) | 25.44 ( 4194.07 ) | 4.63 ( 2.96 ) |
|  | Off label use | 124 | 0.35 ( 0.29 - 0.42 ) | 0.35 ( 148.13 ) | -1.5 ( -3.16 ) |
|  | Incorrect dose administered | 122 | 1.54 ( 1.29 - 1.84 ) | 1.54 ( 22.94 ) | 0.62 ( -1.05 ) |

Abbreviation: ROR, reporting odds ratio; PRR, proportional reporting ratio; IC, information component; IC025, the lower limit of the 95% CI of the IC; CI, confidence interval; PT, preferred term.
