# Supplementary material for: Safety of lifitegrast: A real-world pharmacovigilance study based on FAERS
Source: PLoS One. 2025 Apr 24;20(4):e0321307. doi: 10.1371/journal.pone.0321307 (PMC12021224; doi:10.1371/journal.pone.0321307)
Supplement: S5 Table — (DOCX) [file pone.0321307.s005.docx]

**S5 Table****. Top 30 most frequent AEs for Lifitegrast at the PT level in patients aged 18 to 65 from the FAERS database.**

| **SOC** | **PT** | **Case number** | **ROR (95%CI)** | **PRR (χ^2^)** | **IC(IC025)** |
| --- | --- | --- | --- | --- | --- |
| Nervous system disorders | Dysgeusia | 191 | 49.02 ( 42.33 - 56.76 ) | 46.53 ( 8396.79 ) | 5.52 ( 3.85 ) |
|  | Headache | 67 | 1.47 ( 1.15 - 1.87 ) | 1.46 ( 9.85 ) | 0.55 ( -1.12 ) |
|  | Taste disorder | 27 | 20.66 ( 14.13 - 30.21 ) | 20.52 ( 498.34 ) | 4.35 ( 2.68 ) |
| General disorders and administration site conditions | Instillation site pain | 187 | 5286.41 ( 4190.95 - 6668.22 ) | 5018.49 ( 364819.59 ) | 10.93 ( 9.26 ) |
|  | Instillation site reaction | 103 | 19905.09 ( 11903.48 - 33285.46 ) | 19349.35 ( 282313.03 ) | 11.42 ( 9.73 ) |
|  | Drug ineffective | 84 | 1.21 ( 0.98 - 1.51 ) | 1.21 ( 3.09 ) | 0.27 ( -1.39 ) |
|  | Feeling abnormal | 43 | 3.13 ( 2.31 - 4.22 ) | 3.1 ( 61.42 ) | 1.63 ( -0.03 ) |
|  | Instillation site erythema | 40 | 3074.84 ( 1991.76 - 4746.88 ) | 3041.51 ( 62273.28 ) | 10.61 ( 8.91 ) |
|  | Instillation site irritation | 37 | 1612.96 ( 1085.56 - 2396.58 ) | 1596.79 ( 39338.57 ) | 10.06 ( 8.37 ) |
|  | Instillation site pruritus | 36 | 5047.9 ( 2988.49 - 8526.48 ) | 4998.65 ( 70122.85 ) | 10.93 ( 9.22 ) |
|  | Condition aggravated | 26 | 1.15 ( 0.78 - 1.69 ) | 1.15 ( 0.52 ) | 0.2 ( -1.46 ) |
|  | Malaise | 22 | 0.87 ( 0.57 - 1.32 ) | 0.87 ( 0.45 ) | -0.21 ( -1.87 ) |
| Injury, poisoning and procedural complications | Overdose | 186 | 18.46 ( 15.92 - 21.4 ) | 17.58 ( 2900.02 ) | 4.13 ( 2.46 ) |
|  | Product dose omission issue | 83 | 4.19 ( 3.37 - 5.22 ) | 4.12 ( 197.16 ) | 2.04 ( 0.38 ) |
|  | Product use issue | 43 | 3.85 ( 2.85 - 5.2 ) | 3.82 ( 89.65 ) | 1.93 ( 0.27 ) |
|  | Product prescribing issue | 23 | 27.99 ( 18.54 - 42.25 ) | 27.82 ( 589.75 ) | 4.79 ( 3.12 ) |
| Eye disorders | Vision blurred | 181 | 24.17 ( 20.81 - 28.08 ) | 23.04 ( 3796.49 ) | 4.52 ( 2.85 ) |
|  | Eye irritation | 157 | 74.08 ( 63.03 - 87.07 ) | 70.97 ( 10601.32 ) | 6.12 ( 4.45 ) |
|  | Eye pain | 98 | 37.27 ( 30.46 - 45.6 ) | 36.3 ( 3329.07 ) | 5.17 ( 3.5 ) |
|  | Ocular hyperaemia | 86 | 36.42 ( 29.37 - 45.15 ) | 35.59 ( 2861.19 ) | 5.14 ( 3.47 ) |
|  | Lacrimation increased | 48 | 29.65 ( 22.27 - 39.47 ) | 29.28 ( 1299.59 ) | 4.86 ( 3.19 ) |
|  | Eye discharge | 47 | 93.32 ( 69.7 - 124.94 ) | 92.14 ( 4118.92 ) | 6.49 ( 4.82 ) |
|  | Eye swelling | 45 | 20.2 ( 15.04 - 27.13 ) | 19.97 ( 806.16 ) | 4.31 ( 2.64 ) |
|  | Eye pruritus | 42 | 20.89 ( 15.4 - 28.35 ) | 20.67 ( 781.44 ) | 4.36 ( 2.69 ) |
|  | Visual impairment | 30 | 4.37 ( 3.05 - 6.26 ) | 4.34 ( 77.27 ) | 2.12 ( 0.45 ) |
|  | Eye disorder | 25 | 17.25 ( 11.63 - 25.6 ) | 17.14 ( 378.18 ) | 4.09 ( 2.43 ) |
| Product issues | Product quality issue | 48 | 10.36 ( 7.79 - 13.77 ) | 10.23 ( 399.14 ) | 3.35 ( 1.68 ) |
| Infections and infestations | Sinusitis | 32 | 4.19 ( 2.96 - 5.93 ) | 4.16 ( 76.93 ) | 2.06 ( 0.39 ) |
| Immune system disorders | Hypersensitivity | 43 | 3.84 ( 2.84 - 5.18 ) | 3.8 ( 89.04 ) | 1.93 ( 0.26 ) |
| Gastrointestinal disorders | Nausea | 35 | 0.67 ( 0.48 - 0.94 ) | 0.68 ( 5.46 ) | -0.56 ( -2.23 ) |

Abbreviation: ROR, reporting odds ratio; PRR, proportional reporting ratio; IC, information component; IC025, the lower limit of the 95% CI of the IC; CI, confidence interval; PT, preferred term.
