# Supplementary material for: Safety of lifitegrast: A real-world pharmacovigilance study based on FAERS
Source: PLoS One. 2025 Apr 24;20(4):e0321307. doi: 10.1371/journal.pone.0321307 (PMC12021224; doi:10.1371/journal.pone.0321307)
Supplement: S6 Table — (DOCX) [file pone.0321307.s006.docx]

**S6 Table. Top 30 most frequent AEs for Lifitegrast at the PT level in patients aged over 65 from FAERS the database.**

| **SOC** | **PT** | **Case number** | **ROR (95%CI)** | **PRR (χ^2^)** | **IC(IC025)** |
| --- | --- | --- | --- | --- | --- |
| Eye disorders | Vision blurred | 447 | 55.97 ( 50.71 - 61.79 ) | 50.87 ( 21185.8 ) | 5.62 ( 3.96 ) |
|  | Eye irritation | 282 | 89.96 ( 79.51 - 101.77 ) | 84.75 ( 22121.59 ) | 6.33 ( 4.66 ) |
|  | Eye pain | 129 | 34.57 ( 28.96 - 41.25 ) | 33.67 ( 4003.44 ) | 5.04 ( 3.38 ) |
|  | Lacrimation increased | 108 | 44.52 ( 36.69 - 54.02 ) | 43.54 ( 4366.38 ) | 5.4 ( 3.74 ) |
|  | Ocular hyperaemia | 90 | 30.46 ( 24.67 - 37.59 ) | 29.91 ( 2467.49 ) | 4.88 ( 3.21 ) |
|  | Visual impairment | 75 | 7.08 ( 5.63 - 8.9 ) | 6.99 ( 383.83 ) | 2.8 ( 1.13 ) |
|  | Eye pruritus | 45 | 21.26 ( 15.82 - 28.58 ) | 21.07 ( 849.06 ) | 4.38 ( 2.71 ) |
|  | Eye discharge | 43 | 59.59 ( 43.88 - 80.92 ) | 59.06 ( 2362.99 ) | 5.83 ( 4.16 ) |
|  | Eye swelling | 37 | 16.45 ( 11.89 - 22.78 ) | 16.34 ( 527.25 ) | 4.02 ( 2.35 ) |
|  | Photophobia | 35 | 38.66 ( 27.61 - 54.14 ) | 38.39 ( 1243.41 ) | 5.23 ( 3.56 ) |
|  | Eye disorder | 28 | 10.05 ( 6.92 - 14.59 ) | 10 ( 225.36 ) | 3.31 ( 1.65 ) |
|  | Ocular discomfort | 27 | 34.63 ( 23.62 - 50.76 ) | 34.44 ( 857.37 ) | 5.07 ( 3.41 ) |
| General disorders and administration site conditions | Instillation site pain | 352 | 2958.47 ( 2477.52 - 3532.78 ) | 2742.31 ( 343891.99 ) | 9.93 ( 8.26 ) |
|  | Instillation site reaction | 274 | 27585.28 ( 16648.83 - 45705.77 ) | 26015.91 ( 393274.11 ) | 10.49 ( 8.81 ) |
|  | Drug ineffective | 122 | 1.79 ( 1.49 - 2.14 ) | 1.77 ( 41.03 ) | 0.82 ( -0.85 ) |
|  | Instillation site irritation | 61 | 845.56 ( 617.9 - 1157.11 ) | 834.86 ( 32787.54 ) | 9.07 ( 7.39 ) |
|  | Instillation site lacrimation | 45 | 3136.71 ( 1882.3 - 5227.08 ) | 3107.41 ( 45886.3 ) | 10 ( 8.29 ) |
|  | Condition aggravated | 39 | 1.7 ( 1.24 - 2.33 ) | 1.69 ( 11.1 ) | 0.76 ( -0.91 ) |
|  | Instillation site pruritus | 35 | 1093.06 ( 707.71 - 1688.24 ) | 1085.13 ( 22114.11 ) | 9.31 ( 7.61 ) |
|  | Instillation site erythema | 35 | 677.97 ( 454.92 - 1010.39 ) | 673.05 ( 16276.27 ) | 8.87 ( 7.18 ) |
| Nervous system disorders | Dysgeusia | 148 | 24.72 ( 20.96 - 29.15 ) | 23.99 ( 3213.87 ) | 4.56 ( 2.9 ) |
|  | Headache | 70 | 1.95 ( 1.54 - 2.46 ) | 1.93 ( 31.65 ) | 0.95 ( -0.72 ) |
|  | Dizziness | 35 | 0.81 ( 0.58 - 1.13 ) | 0.81 ( 1.49 ) | -0.3 ( -1.96 ) |
| Product issues | Product quality issue | 98 | 15.59 ( 12.75 - 19.06 ) | 15.29 ( 1297.99 ) | 3.92 ( 2.25 ) |
|  | Product container issue | 31 | 50.15 ( 35.03 - 71.8 ) | 49.84 ( 1436.59 ) | 5.59 ( 3.92 ) |
| Injury, poisoning and procedural complications | Incorrect dose administered | 39 | 2.44 ( 1.78 - 3.34 ) | 2.42 ( 32.68 ) | 1.28 ( -0.39 ) |
|  | Product use complaint | 33 | 19.26 ( 13.65 - 27.18 ) | 19.13 ( 560.32 ) | 4.24 ( 2.57 ) |
|  | Wrong technique in product usage process | 32 | 1.23 ( 0.87 - 1.74 ) | 1.23 ( 1.35 ) | 0.29 ( -1.37 ) |
| Immune system disorders | Hypersensitivity | 28 | 2.99 ( 2.06 - 4.33 ) | 2.97 ( 36.7 ) | 1.57 ( -0.1 ) |
| Gastrointestinal disorders | Nausea | 27 | 0.48 ( 0.33 - 0.7 ) | 0.48 ( 15.29 ) | -1.06 ( -2.72 ) |

Abbreviation: ROR, reporting odds ratio; PRR, proportional reporting ratio; IC, information component; IC025, the lower limit of the 95% CI of the IC; CI, confidence interval; PT, preferred term.
